# Recent advances of small extracellular vesicle biomarkers in breast cancer diagnosis and prognosis

**DOI:** 10.1186/s12943-023-01741-x

**Published:** 2023-02-16

**Authors:** Yujin Lee, Jie Ni, Julia Beretov, Valerie C. Wasinger, Peter Graham, Yong Li

**Affiliations:** 1 St. George and Sutherland Clinical Campuses, School of Clinical Medicine, UNSW Sydney, Kensington, NSW 2052 Australia; 2 Cancer Care Centre, St. George Hospital, Kogarah, NSW 2217 Australia; 3 Anatomical Pathology, NSW Health Pathology, St. George Hospital, Kogarah, NSW 2217 Australia; 4 Bioanalytical Mass Spectrometry Facility, Mark Wainwright Analytical Centre, UNSW Sydney, Kensington, NSW 2052 Australia; 5 School of Medical Science, UNSW Sydney, Kensington, NSW 2052 Australia

**Keywords:** Breast cancer, Extracellular vesicles, Liquid biopsy, Biomarker, Diagnosis, Prognosis

## Abstract

Current clinical tools for breast cancer (BC) diagnosis are insufficient but liquid biopsy of different bodily fluids has recently emerged as a minimally invasive strategy that provides a real-time snapshot of tumour biomarkers for early diagnosis, active surveillance of progression, and post-treatment recurrence. Extracellular vesicles (EVs) are nano-sized membranous structures 50–1000 nm in diameter that are released by cells into biological fluids. EVs contain proteins, nucleic acids, and lipids which play pivotal roles in tumourigenesis and metastasis through cell-to-cell communication. Proteins and miRNAs from small EVs (sEV), which range in size from 50–150 nm, are being investigated as a potential source for novel BC biomarkers using mass spectrometry-based proteomics and next-generation sequencing. This review covers recent developments in sEV isolation and single sEV analysis technologies and summarises the sEV protein and miRNA biomarkers identified for BC diagnosis, prognosis, and chemoresistance. The limitations of current sEV biomarker research are discussed along with future perspective applications.

## Introduction

Breast cancer (BC) is a global public health concern accounting for nearly 30% of female cancers [1]. In 2021, about 284,200 females were newly diagnosed with BC in the US resulting in 43,600 deaths [2, 3]. Early diagnosis of BC is essential for selection of effective treatments and reducing the possibility of cancer metastasis [4]. BC is a heterogenous and dynamic disease with unique somatic mutations accompanied by changes in gene and protein expression. It is classified into distinct subtypes based on estrogen receptor (ER), progesterone receptor (PR), and human epidermal growth factor receptor 2 (HER2) expression. Individual biomarker profiles influence tumour recurrence, drug resistance, and mortality and require different therapeutic approaches [5, 6]. Due to the complexity of BC disease, identification of additional clinical biomarkers is necessary to further stratify patients and provide more information on initial diagnosis as well as the monitoring cancer progression, metastasis, and relapse [7].

Currently, the most common tool for detection, staging, and prognosis of cancer is tissue biopsy. However, a tissue biopsy is difficult to obtain, and tumour molecular and genetic information from the biopsy provides limited information for early detection, screening, and monitoring. Mammography is the only clinically proven imaging method for the early diagnosis of BC, with high potential for false-negative diagnosis and low sensitivity for dense breast tissue [8, 9]. Small lesions are frequently missed and may not be visible by mammograms [10, 11]. Therefore, a significant proportion of detected tumours in women undergoing regular screening have already disseminated, and therefore these patients present with invaded lymph nodes or general metastases at the time of diagnosis. Furthermore, one- to two-year regular mammogram screening does not allow the detection of high growth rate tumours. Although the survival of patients has increased over the last few decades due to screening programs and postoperative adjuvant systemic therapies (i.e., hormone therapy and chemotherapy), many patients die from metastatic relapse [12].

Serum levels of cancer antigen 15–3 (CA 15–3) is the biomarker currently used for BC monitoring. However, CA 15–3 measurements are not helpful in diagnosis, especially in patients with early-stage cancers, and not useful in therapeutic decision-making of patients with BC [13]. Traditional prognostic markers (age at diagnosis, tumour size, hormonal receptor status, tumour grade) are not sufficient for precise risk group discrimination in BC. Therefore, there is an unmet need for discovery of accurate and minimally invasive biomarkers for early detection, prognosis, prediction, monitoring of therapy response, and anticipation of drug resistance in BC patients.

To overcome the challenges of techniques that have traditionally been utilised for identifying and validating clinical biomarkers, analyses based on liquid biopsies have been proposed which focus on circulating tumour cells (CTCs), circulating tumour DNA (ctDNA), tumour-educated platelets, and extracellular vesicles (EVs) in bodily fluids including blood, urine, and saliva [14–17]. Liquid biopsies provide several advantages over collection of tissue biopsies: it is less invasive; it is possible to trace the heterogeneity [18], metastasis, and progression of cancer in a real time [19]; and a patient’s response to treatment can be constantly monitored [20]. Pain and side effects are significantly lower than after collection of tissue biopsies [21, 22].

According to the International Society of Extracellular Vesicles (ISEV), the term “extracellular vesicles” is the appropriate terminology for the heterogeneous populations of vesicles isolated from cell culture supernatants or physiological fluids [23]. The EV populations are defined by their size and biogenesis: exosomes are small-sized EVs (50–150 nm) generated from the fusion of plasma membrane and multivesicular bodies; large-sized microvesicles (large EVs) (100–1000 nm) are formed directly from plasma membrane; and apoptotic bodies (500–5000 nm) are the largest EVs created during programmed cell death [23]. However, there are different classification systems and the groups are not necessarily mutually exclusive. For example, in the ISEV 2018 guideline EV subtypes are classified by physical characteristics such as EV size (small EVs < 200 nm, large EVs > 200 nm), density, and biochemical composition (e.g., CD63, CD81, Annexin A5) [24]. Large EVs (lEVs) have a density 1.10–1.15 g/mL and Cav-1, CK18, and GAPDH expression, while small EVs (sEVs) are characterised by CD9, CD63, CD81, TSG101, Alix, Flotilin-1, and heat-shock proteins (HSPs) and have a density range of 1.05–1.2 g/mL [25]. Based on this classification, this review will focus on sEVs and references that refer to exosomes will fall under this group.

EVs are present in all biofluids including blood [26, 27], urine [28], breast milk, saliva [29], ascites [30], and cerebrospinal fluid [31]. In multicellular organisms, sEVs play a significant role in transmitting biological information from cell to cell and within the tumour microenvironment through various RNAs, proteins, and lipids [32–34]. Several studies have reported that cancer cells release more sEVs than normal cells [35, 36] and their biological cargoes reflect the cell of origin and therefore serve as superior biomarker candidates for disease diagnosis, prognosis, and surveillance [27, 37–39]. Furthermore, genomic and proteomic profiling studies have shown that sEV contents and number are different between BC patients and healthy controls, suggesting they may be a unique source of tumour information [40, 41].

In this review, new techniques that have been developed for sEV isolation and single sEV analysis are summarised. sEVs contain numerous types of biomolecules but the focus here will be on protein and miRNA biomarkers for BC diagnosis and prognosis. The limitations of sEV-based biomarker research will be discussed along with future perspectives of sEVs as a source of novel clinical biomarkers.

## Advantages of sEVs as a biomarker source in BC liquid biopsy

CTCs and ctDNA within liquid biopsies have been studied as diagnostic and prognostic biomarker candidates in various cancers [42–46]. However, the clinical utility of CTCs and ctDNA is facing several critical challenges that need to be resolved. Firstly, CTCs have a short life span (1–2.4 h) [47] and the process of releasing CTCs into the bloodstream is still unclear [48]. The low concentration, dynamically heterogeneous form, and lack of sensitive detection methods of CTCs in bodily fluids make their isolation and characterisation difficult [49, 50]. Analysis of ctDNA is challenging because of low concentration and short half-life, requiring elaborate detection and isolation methods [51].

**Fig. 1:**
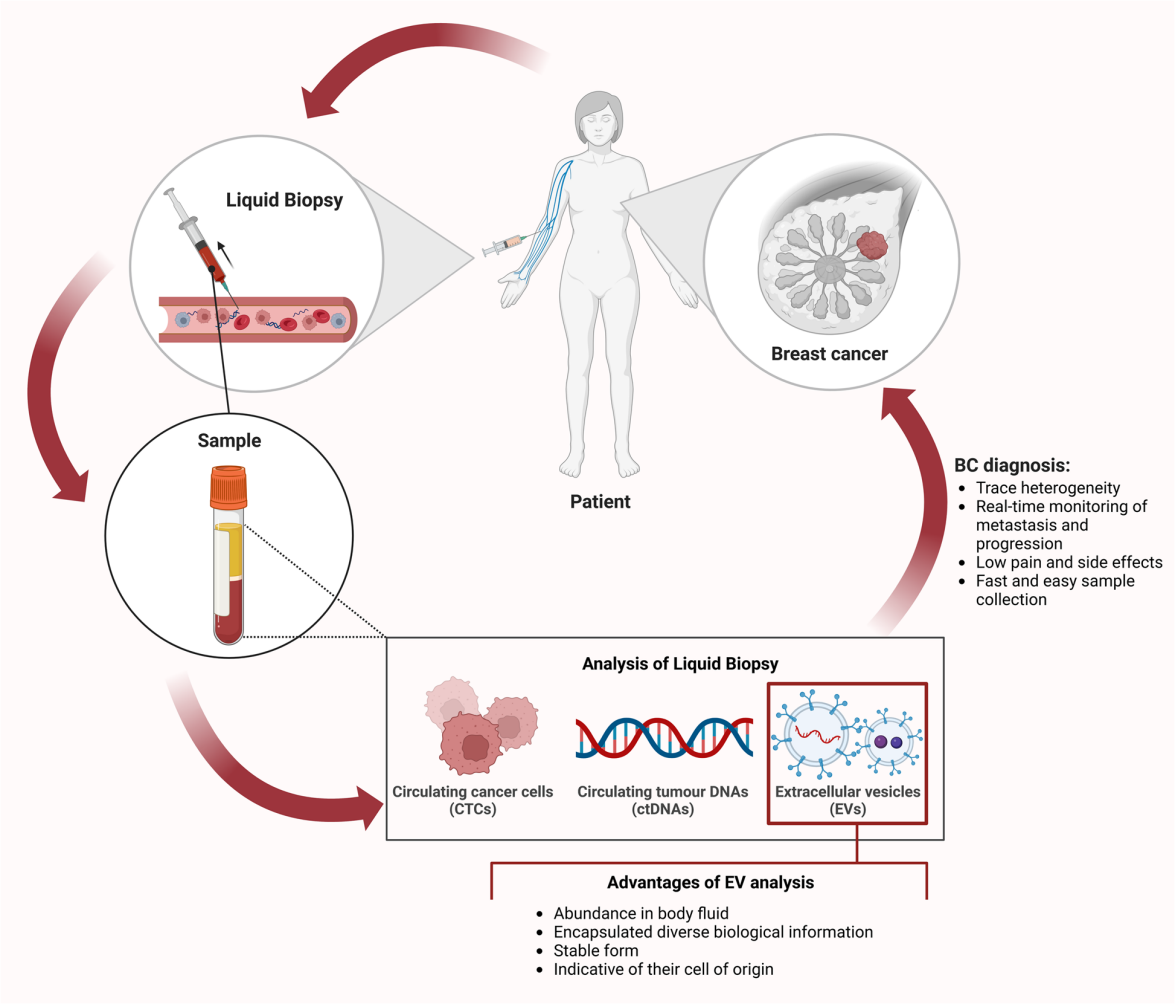
Liquid biopsy for breast cancer (BC) diagnosis. Body fluids are collected from BC patients for liquid biopsy analysis. Circulating tumour cells, circulating tumour DNA, and extracellular vesicles can be detected via liquid biopsy. This approach has several advantages in BC diagnosis such as ability to trace heterogeneity, monitoring of tumour metastasis and progression in a real-time, reduced pain and side effects, and it is a faster and easier process to obtain samples compared to tissue biopsy. Among the analytes detected by liquid biopsy, extracellular vesicles have superior characteristics, for example, abundance in various bodily fluids, stable form with encapsulated bioactive molecules, and reflective of their cell of origin. This figure was created with BioRender.com

Currently, many researchers in the liquid biopsy area have turned their focus on sEVs because of their relatively greater abundance in biofluids, rich biological content from parental cells, stable form [52], and ability to be stored at -80℃ for a long periods of time without significant change in protein contents and morphology [53]. For example, Kirsten rat sarcoma viral oncogene homolog gene (KRAS) and epidermal growth factor receptor (EGFR) DNA mutations detected in plasma sEVs were reported to be sensitive tumour prognostic indicators for pancreatic cancer [54], early-stage non-small-cell lung cancer [55], colorectal cancer, and melanoma [56]. In addition to genetic mutations, the RNAs and proteins from tumour-derived sEVs were shown to have diagnostic value in various cancers such as breast, ovarian, and prostate [38, 57, 58]. For clinical use, the first commercial sEV-based test, ExoDx™ Prostate (IntelliScore), was launched in 2017 and approved by the US Food and Drug Administration (FDA) and uses urinary sEV RNA transcripts. Notably, this test was able to reduce collection of tissue biopsies by 27% in prostate cancer patients [59, 60]. This is evidence that sEVs from liquid biopsy have opened a new pathway for biomarker development which can impact diagnosis, prediction, and treatment of various cancers including BC (Fig. 1).

## Advanced techniques for BC sEV isolation and characterisation

EV research relies on precise and efficient isolation which requires accurate quantitation and high purity. However, heterogeneity in size, density, biochemical composition, and morphology of EVs complicate isolation strategies of specific sEV subpopulations [24, 61, 62]. Various isolation methods have been devised by exploiting a unique characteristic of sEVs such as size, density, composition, morphology, surface proteins, and gauge [63]. The classical sEV separation methods include ultracentrifugation (UC), ultrafiltration, size exclusion chromatography (SEC), polymer precipitation, and immunoaffinity. However, low purity and yield, high cost, and damage to sEVs are the main disadvantages of the traditional isolation methods, and in response, researchers have established more reliable and robust isolation methods. The more recent isolation methods that have emerged include immunoaffinity-based microfluidic chips and magnetic beads which provide simpler and faster detection and isolation of sEVs from cancer patients and with higher efficiency and specificity than the conventional methods [64–68]. Some of the limitations of these approaches include a higher cost and low sEV yield following removal of the affinity agents. Recent reviews discussed the strengths and merits of these approaches [63, 69, 70], and the focus here will centre on newly advanced sEV isolation techniques as it pertains to BC research.

Wang et al. [71] reported isolation of nanoscale vesicles with high yield and purity from plasma of BC patients using microfluidic chips with ciliated micropillars [72]. The Sub-ExoProfile chip is a microfluidic nanodevice with three self-assembled 3D nanopillars to capture CD81, EpCAM, and HER2-specific sEVs and tested in BC cell lines and plasma samples where it was capable of distinguishing HER2^+^ BC and triple-negative BC (TNBC) using a small volume of sample [73]. Additionally, microfluidic chips with filtration approaches such as ExoDIF [74] and ExoID-Chip [75] were validated in BC blood samples, showing high sEV concentration, sensitivity, and selectivity, and they successfully were able to distinguish between BC patients and healthy donors for diagnosis. Most recently, a novel lipid microarray based on supporting lipid membranes carrying antibody CD63 and EpCAM offered a rapid and accurate capture of cancer-specific sEVs in BC cells with minimal sample volume [67]. Despite the effort to develop novel isolation techniques, it is still difficult to apply these methods in a clinical setting due to issues with separation of similar-sized particles, obtaining a high yield of sEVs from a small volume of sample, and high cost.

Because of the high cost of immuno-based isolation methods, researchers have developed alternative approaches that do not rely on antibodies, for example, lipid nanoprobes [76], anion-exchange chromatography [77], and cholesterol-modified magnetic beads [78]. A synthetic peptide, Vn96, was developed that could bind HSPs on the surface of sEVs [79]. Tests showed that this affinity reagent was able to isolate sEVs with reduced background from BC cancer cell lines and plasma samples compared to UC. Moreover, applying Vn96 isolation with TRIzol^©^ extraction in BC cell lines allowed multi-omic analysis of sEV proteins and RNAs on the same samples with a minimal processing time [80]. Zang et al. developed a DNA aptamer-based magnetic technique that targets CD63 on the surface of sEVs [81]. This approach maintained high bioactivity of sEVs and was able to detect increased MUC1^+^ sEVs in plasma samples of BC patients. However, a recent report showed that the DNA aptamer demonstrated low sEV specificity and reproducibility for sEV proteins in clinical settings [82]. Development of higher quality aptamers would require higher cost and more labour [83]. Therefore, method improvement is essential if clinical affinity-based isolation techniques are to be adopted in the future and this requires efficiencies in development and manufacturing cost.

Currently, standardised sEV isolation methods have not been established. The ideal sEV enrichment methodology would be simple, high throughput, and fast with low cost and high purity. However, none of the current isolation methodologies meet these criteria due to the complexity of biological samples and heterogenous nature of EVs. One possibility to overcome these obstacles may be to conjoin two or more isolation techniques [84]. For example, SEC followed by polyethylene glycol (PEG)-based precipitation in BC cell lines was reported to enrich 20- to 200-fold higher concentrations of sEV proteins and in less time than SEC or UC alone [85]. Also, ExoQuick or Total Exosome Isolation kits followed by a half cycle of UC in the serum of BC patients led to significantly purer yields, higher throughput, and with a less time-consuming process [86]. Tayebi et al. developed EV subpopulation sorting using combined electrical and acoustic forces [87]. The purity of sEVs recovered and recovery rate were higher in the combinational approach than in either individual approach. Lastly, clinical grade sEVs from human adipose mesenchymal stromal cells were isolated by a combination of UC and PEG-based isolation techniques and these sEVs were used for aerosol inhalation in lung injury to decrease inflammation [88]. Taken together, continued development of combined isolation techniques may be a viable option for selecting and isolating sEVs, which could aid in downstream analysis and clinical applications.

## Single sEV analysis in BC research

To overcome issues such as heterogeneity, impurities during enrichment, inaccurate quantitation, and damage to sEVs, techniques based on molecular composition of single sEVs have been developed [89–91]. Single particle interferometric reflectance imaging sensor (SP-IRIS) can help overcome limitations such as overestimation of sEV counts and detection size limits, and simultaneously detect and count single sEV particles and analyse relative size distribution [92]. Jung et al. used SP-IRIS to compare several different sEV isolation methods based on protein marker expression such as CD9, CD63, and CD81 and found that Exoquick isolation kits had higher sEV particle concentration compared to exoEasy and UC isolates [93].

Instead of conventional flow cytometry (FCM), researchers have shown that a custom-built high-sensitivity flow cytometer [94], a fluorescence-activated vesicle sorting (FAVS) instrument [95], and a high-resolution flow cytometer (hFCM) [96] improved upon sensitivity and resolution. For example, FAVS was shown to localise EGFR ligand on the outside of sEVs in a BC cell line [97]. Risha et al. used hFCM to verify expression levels of glucose transporter 1 (GLUT-1), glypican 1 (GPC-1), and disintegrin and metalloproteinase domain-containing protein 10 (ADAM10) on the surface of sEVs derived from BC [98].

In addition to these techniques, nano-flow cytometry (nFCM) can analyse single sEVs below diameters of 40 nm with low background signal, enhanced signal integration, and high sensitivity [99, 100]. This method can quantify individual sEVs without a surface anchoring process and only requires small volumes compared to conventional FCM [101]. Recent studies demonstrated that nFCM can capture heterogeneously distributed cargoes across different EV subpopulations in colorectal cancer cell lines and Expi293F cells [102, 103]. In BC plasma samples, Salmond et al. used nFCM to distinguish sEVs derived from platelets and protein aggregations depending on their size, markers, and detergent treatment, showing the relatively high sensitivity of nFCM. Also, the majority of CD9^+^ and mammaglobin^+^ sEVs in human BC platelets were successfully identified using nFCM [104]. Expression of tissue factor (TF/F3) and EGFR among EV subpopulations from a BC cell line were mapped by nFCM [105].

A method for super-resolution microscopy, called photoactivated localisation microscopy/stochastic optical reconstruction microscopy (PALM/STORM), has also been utilised for the detection of single sEVs (< 100 nm) in BC cell lines. Results showed that a large portion of sEVs taken up by BC cell lines were localised inside lysosomes, providing evidence that sEVs can be endocytosed by recipient cells and transported into lysosomes for further degradation [106].

The droplet-based single-exosome-counting enzyme-linked immunoassay (droplet digital ExoELISA) was used for absolute quantitation of glypican-1^+^ sEVs in the plasma of BC patients by targeting protein expression on single sEVs in droplets [107]. Results showed that glypican-1^+^ sEVs were highly expressed in BC cells compared to benign breast disease and numbers were reduced after surgery, showing good potential of this technique for BC diagnosis. Recently, digital profiling of proteins on individual sEVs (DPPIE) was developed to multiplex detection of proteins on individual sEVs. DPPIE showed higher CD63/EpCAM/MUC1^+^ sEVs in BC plasma compared to healthy donors [108]. Finally, digital droplet PCR (ddPCR) was able to simultaneously quantify four mRNAs in individual sEVs derived from BC plasma samples with high sensitivity, demonstrating a novel method for profiling PR, ER, and HER2 subtypes [109].

**Fig. 2:**
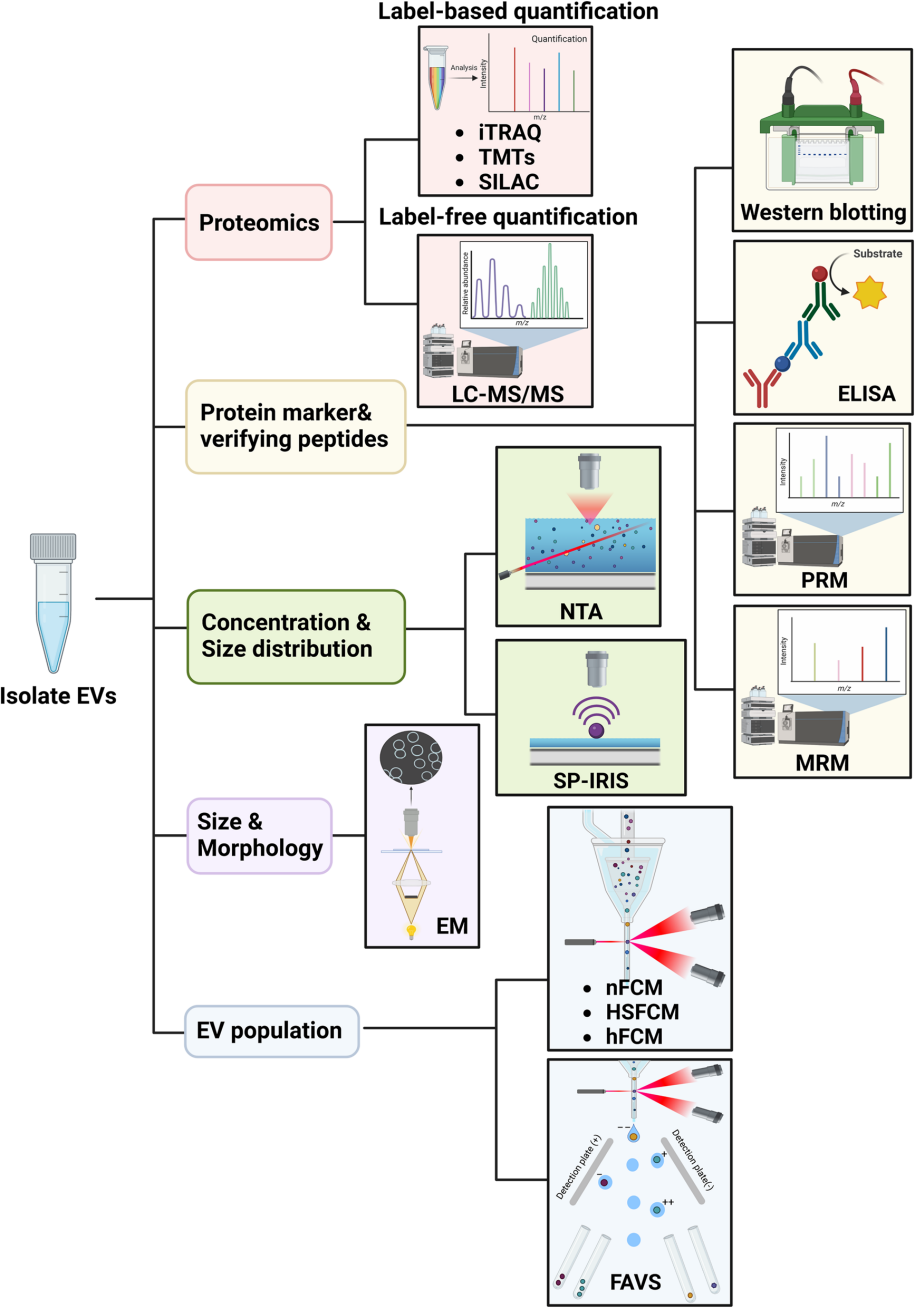
Small extracellular vesicle (sEV) detection and characterisation. sEVs isolated from breast cancer (BC) blood samples and cell lines can be characterised by size, morphology, concentration, and sEV markers. sEV size distribution and concentration can be analysed by nanoparticle tracking analysis (NTA) and single particle interferometric reflectance imaging sensor (SP-IRIS). Various types of electron microscopy (EM) can help classify sEVs by size and morphology. Nano flow cytometry (nFCM), high-sensitivity flow cytometry (HSFCM), high-resolution flow cytometry (hFCM), and fluorescence-activated vesicle sorting (FAVS) can sort sEV subpopulations in the isolated samples using various sEV markers. sEV proteins can be profiled by mass spectrometry-based analysis including label-free and label-based quantification. For verifying sEV protein and peptide markers, multiple reaction monitoring (MRM), parallel reaction monitoring (PRM), western blotting (WB), and enzyme-linked immunosorbent assay (ELISA) can be used. This figure was created with BioRender.com

Precise isolation and characterisation of each EV subpopulation is still a formidable challenge [110]. However, advances are ongoing and the advantages of current techniques for analysis of individual sEVs in BC research are summarised in Table 1 and sEV detection, characterisation, and analytical methodologies are shown in Fig. 2. In summary, single sEV detection holds great promise for early diagnosis and progression monitoring of BC and personalised medicine.

**Table 1:** Summary of small extracellular vesicle analysis methods in breast cancer research

Detection technique	Detection material	Advantage	Reference
SP-IRIS	Concentration and size distribution	High throughput and sensitive detection of single EVs	[92]
HSFCM	Specific EV population and size distribution	Multiparameter analysis of single EVs as small as 40 nm and analysis 10 000 particles per minute	[94]
FAVS	Specific EV population	High sensitivity, speedy detection, and elimination of background noise	[95, 111]
hFCM	Specific EV population	Low detection limit and multiparameter qualitative analysis	[96]
nFCM	Specific EV population	Analysation of EVs smaller than 40 nm, high throughput, and high resolution with multiparametric scattered light to detect individual EVs	[99, 100, 112]
dSTORM	Three-dimensional shape of sEVs	Imaging resolution of approximately 20 nm, inexpensive equipment	[106, 113]
Droplet digital ExoELISA	Absolute quantification of sEVs	Detection of maximum 5 sEVs per microliter of samples with high sensitivity and specificity, absolute quantification of targeting sEVs	[107]
DPPIE	Multi-quantification of surface protein of sEVs	Analysation of muti-protein expression in individual sEVs with high sensitivity and small volume of samples, and no purification step required	[108]
ddPCR	Quantify RNAs from single sEVs	Absolute quantification of rare targets	[114]

*Abbreviations*: *ddPCR* droplet digital PCR, *DPPIE* Digital profiling of proteins on individual sEVs, *Droplet digital ExoELISA* Droplet-based single-exosome-counting enzyme-linked immunoassay, *dSTORM* direct stochastic optical reconstruction microscopy, *EV* Extracellular vesicle, *FAVS* Fluorescence-activated vesicle sorting, *hFCM* high-resolution flow cytometer, *HSFCM* High-sensitivity flow cytometer, *nFCM* nano-flow cytometer, *SP-IRIS* Single particle interferometric reflectance imaging sensor

## sEV protein biomarkers and post translational modifications (PTMs) in BC diagnosis, prognosis, and chemoresistance

Early proteomic approaches for sEV biomarker research evolved from western blotting (WB) to ELISA, one-dimensional electrophoresis, and liquid chromatography with tandem mass spectroscopy (LC–MS/MS), however, these methods are limited by low acquisition rate, limited throughput, low sensitivity, and high cost [115, 116]. Unlike ELISA and WB which quantify single proteins, improved MS-based technology allows global analysis by detection of thousands of sEV proteins in one experiment [32]. Current LC–MS/MS-based quantitative proteomics are categorised into two approaches: label-free and label-based quantification. Label-free quantification is a commonly used proteomic approach to identify sEV proteins in cells [117] and various body fluids samples such as plasma [118], serum [119], saliva [120], urine [121], and cerebrospinal fluid [122, 123]. However, it is among the lowest for throughput among MS-based methods [124] and has low reproducibility because of multi-step sample processing [125]. One researcher reported improved throughput by using label-free LC–MS/MS combined with the absolute protein expression (APEX) algorithmic tool in cancer-derived sEV protein detection, showing a more time-effective and less laborious detection process for cancer biomarker discovery [126].

For label-based quantification, isobaric tags for absolute and relative quantification (iTRAQ), tandem mass tags (TMTs), and stable isotope labelling of amino acids (SILAC) are examples of label-based quantification that have been applied to sEV research in various diseases including BC [127–132]. However, these techniques have limitations in terms of high cost, low throughput, and lack of specificity during ion selection [133, 134]. To increase throughput and specificity, Ting et al. utilised a multiplex approach using TMT reagents which requires an additional isolation and fragmentation event (MS3) [135]. For the purpose of identifying potential biomarkers in BC diagnosis and monitoring of disease progression, Clark et al. combined TMTs with support vector machine analytics to detect 251 sEV proteins in BC cells [136].

One of the modern techniques used for sEV protein biomarker validation is multiple reaction monitoring (MRM), which provides a more accurate quantification of low abundant peptides than other targeted methods and without the necessity for expensive antibodies [137, 138]. Several studies have used MRM to obtain detailed expression profiles of sEV proteins and to identify potential biomarkers such as HSP90 and syndecan-1 in bladder cancer, and annexin A2 and clusterin in Alzheimer's disease [139–141]. However, the precise measurements made by MRM can only be applied for small peptides, not for multiple peptides in the same protein [142], which may limit its clinical translation as an assay.

The parallel-reaction monitoring (PRM) technique is similar to MRM, but quantitation is performed with MS2 in a high-resolution mass spectrometer. It has higher throughput, high sensitivity, and less preparatory time in peptide identification, and it is adequate for the identification of PTMs [143, 144]. PRM was used to validate selected sEV proteins from a global profiling study [129]. Additionally, it was utilised to detect elevated sEV phosphoproteins with a potential diagnostic value such as GTPase-activating protein subunit alpha-2 (RALGAPA2), cGMP-dependent protein kinase1 (PKG1), and tight junction protein 2 (TJP2) in BC patients [145]. Proteomic techniques used for sEV protein biomarker detection are shown in Fig. 2.

### sEV protein biomarkers in BC diagnosis

Proteins contained within sEVs consist of almost half of the human proteome and are reflective of the cell types of origin, suggesting an ideal choice for disease-specificity and biomarker discovery [146]. Accumulating evidence indicates sEV protein biomarkers play an important role in BC diagnosis. Lee et al. detected 270 sEV proteins in invasive BC cell lines using LC–MS/MS and validated EGF-like repeat and discoidin I-like domain containing protein 3 (EDIL3) as a diagnostic biomarker that is correlated with metastasis [147]. Another study profiled 241 uniquely expressed sEV proteins in several BC cell lines, demonstrating fibronectin (FN) as a diagnostic biomarker candidate specifically for distinguishing between ER^+^ and ER^−^ BC [40, 148]. Using a reverse phase protein microarray, up-regulated focal adhesion kinase (FAK) and mitogen-activated protein kinase 1 (MEK1) in plasma sEVs were identified as candidate biomarkers for BC diagnosis [40]. With micro FCM, CD47 was found to have relatively lower abundance levels in sEVs isolated from serum of BC patients compared to healthy controls [149]. Risha et al. profiled 726 uniquely expressed proteins in TNBC cells by nano LC–MS/MS and identified three potential biomarkers located on the membrane surface of sEVs (GPC-1, glucose GLUT-1, and ADAM10), and up-regulated when compared to a non-tumourigenic epithelial breast cell line [98]. A multiplexed cantilever array was used to profile GPC-1 in sEVs released from BC cell lines, showing high sensitivity and throughput in real-time acquisition [150].

Recently, several novel sEV screening methods including microfluidic chips [151], surface enhanced Raman scattering nanotags [152], and DNA aptamer-mediated microfluidics [153] have demonstrated simple and time-saving ways to profile sEV EpCAM and HER2 proteins for diagnosis of HER2^+^ BC. Cumulatively, these results demonstrate the diagnostic value of sEV protein biomarkers in BC, however, further validation in large studies of independent clinical samples is required to confirm their clinical significance.

### sEV protein biomarkers in BC prognosis

Determination of the prognostic value of sEV protein biomarkers is an important area of BC research. Higher levels of sEV annexin A2 was reported using a combination of two-dimensional gel electrophoresis and matrix-assisted laser desorption/ionisation time-of-flight (MALDI-TOF) MS in BC cell lines and plasma of BC patients [154]. Also, sEV annexin A2 in serum was shown to have a prognostic value with a positive correlation to tumour grade of TNBC and poor survival [155]. In BC patients treated with neoadjuvant chemotherapy, elevated nerve growth factor (NGF) within serum-derived sEVs was related to poor survival outcomes, suggesting NGF in sEVs was an independent prognostic factor for overall survival [156]. The level of insulin-like growth factor receptor β subunit (IGFRβ) in plasma sEVs [40] and CD82 in serum sEVs [157] was found to gradually increase with BC grade, showing the potential of these protein biomarkers for monitoring BC progression or prediction of disease prognosis. In the earliest stage of BC, up-regulated developmental endothelial locus-1 (Del-1) in plasma sEVs was identified as a potential prognostic indicator by LC–MS/MS and ELISA [158]. Survivin was elevated in serum sEVs from early-stage BC and TNBC patients, and its splice variants were inversely correlated to tumour grade in BC [159], suggesting the expression of survivin and its variants might be a potential indicator of BC progression. Interestingly, urinary sEVs from early-stage BC patients had significantly higher levels of matrix metalloproteinase-1 (MMP-1)/CD63 than healthy control subjects, showing a high sensitivity for primary screening of early-stage BC cases [160].

### sEV protein biomarkers in predicting BC chemoresistance

Chemoresistance is a major challenge for BC therapy and prediction of its occurrence can help guide treatment and improve prognosis. Serum from HER2^+^ BC patients was reported to harbour sEVs with elevated levels of HER2 [161]. Furthermore, trastuzumab was found to bind to sEV HER2 in treated patients and likely contributed to resistance to this drug. In addition, unique expression profiles of 51 sEV proteins was reported in HER2^+^ BC cells treated with trastuzumab and these proteins had functional roles related to organelle organisation, cytokinesis, and response to lipids [162]. This study highlighted the role of nano-ultra-HPLC–MS/MS in identifying potential biomarker candidates for predicting trastuzumab chemoresistance. Durcker et al. observed that trastuzumab treatment led to elevated levels of sEV PERP, ITB1, GNAS2, and GNA13 proteins in HER2^+^ and trastuzumab-sensitive BC cells, but not in the resistant cells [163]. Further evidence showed that these proteins were also elevated in plasma sEVs of HER2^+^ BC patients treated with trastuzumab therapy, however, a larger patient cohort is required for verification [163].

Several lines of research suggested that sEV-mediated intercellular cargo delivery from chemoresistant BC cells to sensitive cells might induce a chemoresistant phenotype in the acceptor cells, including GSTP1 [164], UCH-L1 [165], and transient receptor potential channel 5 (TrpC5) [166] proteins in adriamycin-resistant BC cells and serum. In the same way, high abundance of ABCG2/breast cancer resistance protein (BCRP) in plasma sEVs isolated from anthracycline-taxane-based neoadjuvant chemotherapy-resistant BC patients [167], and annexin A6 in gemcitabine-resistant BC cells was found to transfer to chemo-sensitive BC cells via sEVs and induce chemoresistance [168]. During this transfer, annexin A6 derived from gemcitabine-resistant BC cells induced the inhibition of EGFR ubiquitination and degradation [168, 169]. Kavanagh et al. demonstrated that therapeutic-induced senescent (TIS) TNBC cells by paclitaxel (PTX) had 142 significantly elevated sEV proteins such as ATPases, annexins, tubulins, integrins, and Rabs that are associated with cell proliferation, ATP depletion, apoptosis, and insoluble senescence-associated secretory phenotype [170]. This data shows the potential of TNBC TIS as a source of predictive biomarkers as well as support for chemotherapeutic challenge. Thus, sEV protein biomarkers are an important source for predicting BC chemoresistance in the clinical setting and future development of personalised medicine.

### PTMs as BC sEV protein biomarkers

Most proteins contain dynamic PTMs which affect function and stability, and PTMs are known to be integral to sEV formation and sEV-related biological processes [171]. For example, Aguilar et al. detected about 10,000 phosphopeptides and 1,500 N-glycopeptides in a small volume of human plasma-derived sEVs [172]. Several studies have addressed the possibility of phosphorylation and glycosylation of sEV proteins as cancer biomarkers, for example, phospho-EGFR in head and neck cancer [173], phospho-AKT and -ERK1/2 in non-small-cell lung cancer [174], and EGFR-specific N-glycan in colorectal cancer [175].

Although recent studies have profiled phosphoproteins in BC, research that specifically targets sEV phosphoproteins in BC is very limited. A few studies showed evidence that sEV phosphoproteins may have diagnostic value for BC. For example, 144 up-regulated phosphoproteins in plasma sEVs of BC patients were identified by LC–MS/MS and phosphorylation of RALGAPA2, PKG1, and TJP2 were validated by PRM, therefore these phosphoproteins are candidate sEV biomarkers for BC diagnosis [145]. Moreover, in TNBC and HER^+^ BC, sEVs contained hyperphosphorylated receptor tyrosine kinase, non-receptor tyrosine kinase, and MAP kinase, and the downstream signalling pathways of these kinases are related to migration and angiogenesis. It was postulated that BC-derived sEVs can transfer hyperphosphorylated proteins and activate intracellular signalling pathways related to metastasis in recipient cells [176].

For glycosylation PTMs, LC–MS/MS analysis showed the differential expression of 77 glycoproteins in sEVs from plasma of BC patients compared to healthy controls, of which 20 were up-regulated [177]. Elevated sEV glycoproteins include lymphocyte antigen 6 complex locus protein G6f (LY6G6F), von Willebrand factor (VWF), CD147/basigin (BSG), complement C1q subcomponent subunit A (C1QA), and angiopoietin-1 (ANGPT1/Ang1) [177]. Moreover, highly abundant glycosylated sEV proteins glycoprotein 130 in BC cells and CD147 in BC patient serum showed a diagnostic value for BC [178, 179]. Glycoprotein 130 can transfer to macrophages via BC-derived sEVs, leading to phosphorylation of transcription factor STAT3 and induction of tumourigenesis-associated genes IL-6 and IL-10 [179], suggesting the potential of both glycoprotein 130 and CD147 as biomarkers for BC progression monitoring.

sEVs have been reported to enhance the expression of P-glycoprotein (P-gp) in recipient cells. For example, sEVs from adriamycin-resistant BC cells were shown to transfer UCH-L1, activate the MAPK/ERK signalling pathway, and up-regulated P-gp [166]. Similarly, TrpC5 was also delivered to adriamycin-sensitive cells by sEVs but stimulated P-gp expression through the translocation of transcription factor, T-cells isoform c3 (NFATc3) [165]. In PTX-induced TNBC TIS, sEV glycoprotein profiling identified elevated P-gp, CD44, galectin-3, and glycogenin-1, suggesting that altered abundance of glycoproteins in sEVs could be a tool to evaluate the treatment efficacy of TNBC cells that are amenable to PTX chemotherapy [170, 180].

**Fig. 3:**
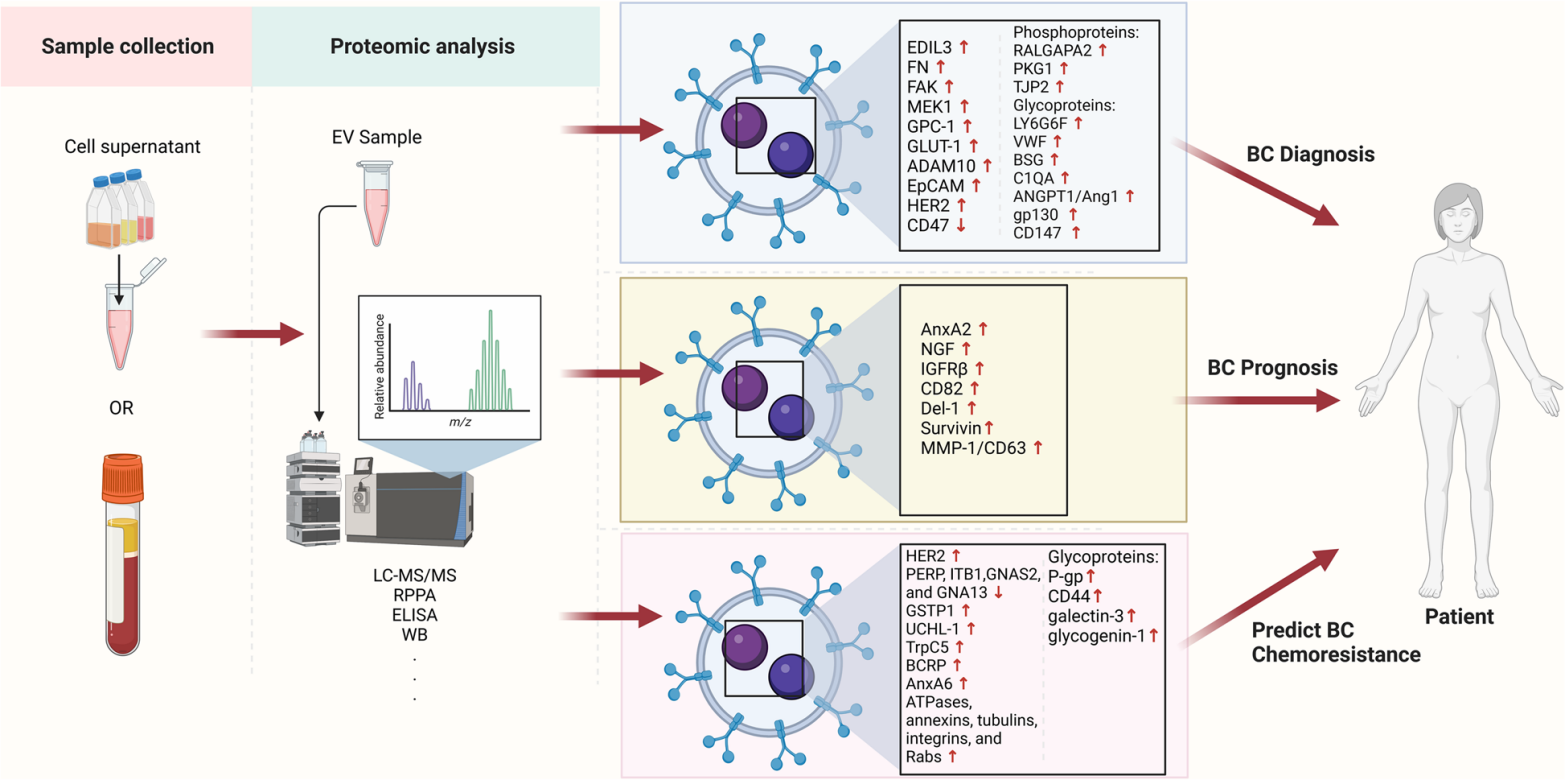
Small extracellular vesicle (sEV) protein biomarkers, including post-translational modifications (PTMs), in breast cancer (BC) diagnosis, prognosis, and chemoresistance. sEVs are collected from cell supernatants and blood samples of BC patients. Isolated sEVs are profiled by proteomic strategies such as liquid chromatography with tandem mass spectrometry (LC–MS/MS) and reverse phase protein microarray (RPPA) and validated by sEV protein detection methods including enzyme-linked immunosorbent assay (ELISA) and western blotting (WB). Various up- or down-regulated proteins in BC sEV samples were identified and can be used in BC diagnosis, prognosis, and prediction of BC chemoresistance. This figure was created with BioRender.com

More research is required to verify PTMs as BC biomarkers. Moreover, high resolution methods for profiling cancer-specific and dynamic phosphorylation and glycosylation of sEV proteins as well as development of PTM-specific antibodies are required for further validation. The role of potential sEV protein biomarkers, including PTMs, in BC diagnosis, prognosis, and chemoresistance are summarised in Fig. 3 and Table 2.

**Table 2:** Small extracellular vesicle protein biomarkers, including post-translational modifications, identified in breast cancer diagnosis, prognosis, chemoresistance

Biomarker	Source	Purpose	Expression	Feature	Technology	Reference
EDIL3	Cells	Diagnosis	↑	Related to invasion ability	LC–MS/MS	[147]
FN	Cells/plasma	Diagnosis	↑	Highest expression in ER^−^ BC followed by ER^+^ and healthy control	LC–MS/MS	[40, 148]
FAK	Plasma	Diagnosis	↑	N/A	RPPA	[40]
MEK1	Plasma	Diagnosis	↑	N/A	RPPA	[40]
CD47	Serum	Diagnosis	↓	N/A	Micro FCM	[149]
GPC-1	Cells	Diagnosis	↑	Located on cell surface, related to proliferation	Nano-LC–MS/MS Sandwich cantilever assay	[98, 150]
GLUT-1	Cells	Diagnosis	↑	Located on the cell surface related to BC migration	Nano-LC–MS/MS	[98]
ADAM10	Cells	Diagnosis	↑	Located on the cell surface	Nano-LC–MS/MS	[98]
EpCAM	Cells/plasma	Diagnosis	↑	Overexpressed in various cancers Diagnosis for HER2^+^ BC	Microfluidic chips SERS nanotags DNA aptamers mediated- microfluidic	[151–153]
HER2	Cells/plasma	Diagnosis Chemoresistance	↑	Diagnosis for HER2^+^ BC Related to trastuzumab resistance	Microfluidic chips SERS nanotags DNA aptamers mediated- microfluidic nano-ultra-HPLC–MS/MS	[151–153, 161, 162]
RALGAPA2, PKG1 & TJP2	Plasma	Diagnosis	↑	Phosphoproteins	LC–MS/MS PRM	[145]
LY6G6F, VWF, BSG, C1QA & ANGPT1/Ang1	Plasma	Diagnosis	↑	Glycoproteins	LC–MS/MS	[177]
Glycoprotein 130	Cells	Diagnosis	↑	Glycoprotein	WB	[179]
CD147	Serum	Diagnosis	↑	Glycoprotein	LC–MS/MS WB	[178]
Annexin A2	Cells/serum	Prognosis	↑	Related to poor survival of BC	MALDI-TOF MS	[154, 155]
NGF	Serum	Prognosis	↑	Related to poor survival of BC who undergo neoadjuvant chemotherapy	ProcartaPlex immune-related panels	[156]
IGFRβ	Plasma	Prognosis	↑	Higher expression in later-stage BC	RPPA	[40]
CD82	Serum	Prognosis	↑	Higher expression in later-stage BC	ELISA	[157]
Del-1	Plasma	Prognosis	↑	N/A	LC–MS/MS ELISA	[158]
Survivin	Serum	Prognosis	↑	High expressed in early-stage BC and TNBC Survivin splice variants negatively correlated with tumour grade	ELISA WB	[159]
MMP-1/CD63	Urine	Prognosis	↑	Higher expression in early-stage BC	WB	[160]
PERP, ITB1, GNAS2 & GNA13	Cells/plasma	Chemoresistance	↓	Higher expressed in HER2^+^ and trastuzumab-sensitive BC	LC–MS/MS	[163]
GSTP1	Cells/serum	Chemoresistance	↑	Higher expressed in adriamycin-resistance BC	FCM WB	[164]
UCHL-1	Cells/serum	Chemoresistance	↑	Higher expressed in adriamycin-resistance BC	FCM WB	[165]
TrpC5	Cells/serum	Chemoresistance	↑	Higher expressed in adriamycin-resistance BC	FCM RT-PCR	[166]
BCRP	Plasma	Chemoresistance	↑	Higher expressed in TEC therapy resistance BC	FCM RT-PCR	[167]
Annexin 6	Cells	Chemoresistance	↑	Higher expressed in gemcitabine resistance BC	Isobaric peptide labelling LC–MS/MS, WB	[168]
ATPases, annexins, tubulins, integrins & Rabs	Cells	Chemoresistance	↑	Higher expressed in TIS cells	LC–MS/MS WB	[170]
P-gp, CD44, galectin-3 & glycogenin-1	Cells	Chemoresistance	↑	Glycoproteins PTX chemotherapy-resistance in TNBC	LC–MS/MS	[170, 180]

*Abbreviations*: *BC* Breast cancer, *ELISA* Enzyme-linked immunosorbent assay, *ER* Estrogen receptor, *FCM* Flow cytometry, *LC–MS/MS* Liquid chromatography with tandem mass spectrometry, *N/A* Not applicable, *PRM* Parallel-reaction monitoring, *PTX* Paclitaxel, *RPPA* Reverse phase protein microarray, *RT-PCR* Reverse transcriptase-polymerase chain reaction, *SERS* Surface enhanced Raman scattering, *TEC* The anthracycline-taxane-based neoadjuvant chemotherapy (docetaxel, epirubicin, and cyclophosphamide), *TIS* Therapeutic induced senescent, *TNBC* Triple negative breast cancer, *UHPLC* Ultra-high performance liquid chromatography, *WB* Western blotting

### Limitations of sEV protein biomarkers in cancer research

MS-based proteomics is the most common method for profiling sEV protein biomarkers in cancer. Although better enrichment strategies for sEVs and more specificity for accurate protein identification by MS are required, current sEV isolation methods attempt to find a balance between purity and abundance which are inversely related. Askeland et al. recently compared three sEV isolation techniques such as UC, SEC, and peptide-affinity precipitation from human plasma, and found all isolates still had contaminations which required additional steps for higher purity but consequently led to a lower yield [82]. In addition, protein contents of sEVs were found to vary depending on the isolation method. For example, when SEC, UC, and PEG-based isolation methods combined with SEC were compared, all showed different relative quantities of sEV proteins [85]. In a comparison study between SEC, PEG-based isolation, and protein organic solvent precipitation (PROSPR), common sEV markers such as CD9, CD63 and CD81 were only detected in sEVs isolated from SEC [181]. Moreover, protein precipitation with acetone in the PROSPR methodology may have led to peptide modification when glycine is the second residue. Since up to 6% of peptides contain glycine as the second residue, this could lead to consequential artifacts in the data [182, 183]. The ISEV guideline has advocated for a universal protocol for obtaining pure sEVs that are compatible with proteomic analysis [184].

Selection of the enzymatic digestion procedure in sample preparation steps which involve contaminant removal for MS-based analysis, including in-gel, in-solution, and filter-aided digestion, affects the identification yield of sEV peptides. When in-solution digestion is used, the profile of sEV proteins varies depending on the choice of sEV isolation method as well as the detergents used for solubilising sEV proteins. For example, using nano LC–MS/MS, Risha et al. identified 986 sEV proteins isolated by UC and digestion in the presence of *n*-dodecyl β-d-maltoside (DDM) detergent, and this total was higher compared to use of other detergents (Triton X-100, Digitonin) and isolation methods (ultrafiltration combined with UC or ExoQuick®) [98]. In-gel digestion is likely the most popular method but can lead to heavy keratin contamination. Xu et al. suggested the use of an electrostatic eliminator to solve the contamination problem, but it was not completely eliminated [185]. Several studies introduced modified filter-aided digestion protocols with sodium dodecyl sulfate detergents [186] and multiple enzyme-filter aided sample preparation (MED-FASP) [187, 188] for LC–MS/MS analysis, showing the highest yield of sEV peptide and protein identifications and with the cleanest MS spectra from blood samples. However, because of its time-consuming and labour-intensive processes, translation to clinical settings remains a challenge.

Several investigations reported that sEVs isolated from blood samples were contaminated with non-vesicular entities such as lipoproteins and protein aggregates [189, 190]. Sodar et al. showed that lipoprotein morphology was similar to sEVs and there were tenfold more lipoprotein particles compared to sEVs in the sEV isolates from human blood which are likely contributing factors to contamination. The authors further postulated that any of the known sEV isolation methods cannot separate lipoproteins from the isolates [191]. However, combining isolation methods can help to reduce contamination. Utilising SEC with a density cushion significantly reduced lipoprotein particles in human plasma samples [192]. The sequential use of PEG precipitation, linear iodixanol density gradients, and SEC filtered out 90% of lipoproteins in plasma samples, however, significant losses of sEVs and arduous sample preparation were limitations for this approach [193].

Considerations for choosing an appropriate sEV isolation technique include the volume of biofluid and the sEV contaminants known to reside in the biofluid. Development of enhanced sEV sample preparation techniques will lead to higher purity, yield, and throughput, as well as better reproducibility and more accurate protein quantification.

## sEV miRNA biomarkers in BC diagnosis, prognosis, and chemoresistance

miRNAs are short single-stranded non-coding RNA molecules about 19–25 nucleotides in length, and can inhibit gene function by directly binding to the 3’-untranslated region of target mRNAs. An RNA profiling study using next-generation sequencing (NGS) found that miRNAs were the most prominent RNA species in sEVs and identified 593 unique miRNAs [194]. In blood, encapsulation of miRNAs in sEVs can provide a stable environment and facilitate long distance cell-to-cell communication. Therefore, tumour-derived sEVs carrying miRNAs that are released into the bloodstream could be a valuable biomarker resource for disease diagnosis, prognosis, and treatment management [116, 195].

**Fig. 4:**
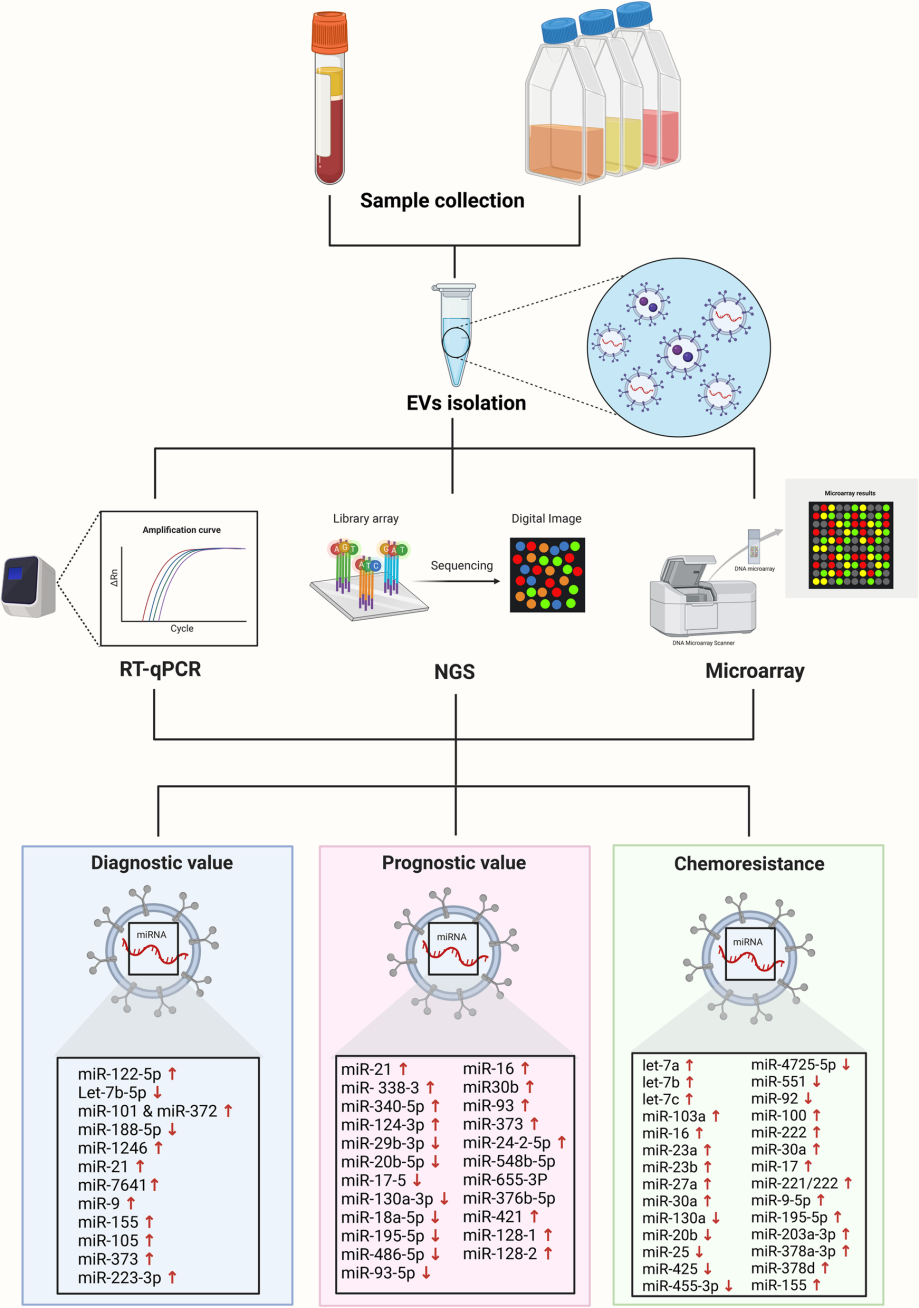
Small extracellular vesicle (sEV) miRNA biomarkers in breast cancer (BC) diagnosis, prognosis, chemoresistance. BC plasma and cell supernatant samples are collected, and the isolated sEVs containing miRNAs are analysed by reverse transcriptase-quantitative polymerase chain reaction (RT-qPCR), next-generation sequencing (NGS), and microarray analysis. The unique sEV miRNA expression profiles of BC samples have value for diagnosis, prognosis, and predicting chemoresistance of BC. This figure was created with BioRender.com

Identification of miRNAs and their targets have been studied by quantitative real-time PCR (qRT-PCR), microarray analysis, and NGS [196–198]. NGS is a second-generation sequencing technique that has followed Sanger sequencing methods. Today, it is the popular method for miRNA profiling with several advantages including high sensitivity and throughput, ability to identify miRNA variants, and low cost [199, 200]. The 4 most widely used NGS systems are the 454 pyrosequencing method, Illumina’s sequencing by synthesis (SBS) technique, Ion Torrent technology, and sequencing by oligonucleotide ligation and detection (SOLiD). The 454, Illumina, and SOLiD methods use four fluorescence dyes for sequence detection. Illumina uses fluorescently labelled reversible terminators that are imaged once a dNTP is added to the sequence, and then cleaved to stop addition of the next base. The SOLiD method does not use DNA polymerase like other methods but rather detects fluorescence when an 8-mer oligonucleotide is added to sequence using a DNA ligase. Instead of fluorescence, Ion Torrent detects proton changes during elongation [201]. Among these four systems, most BC sEV miRNA studies have utilised Illumina sequencing to identify metastatic features related to miRNAs, new therapeutic targets, and biomarker candidates [202–205]. The BC sEV miRNAs identified as a potential diagnostic, prognostic, and chemoresistant biomarkers as well as potential therapeutic targets are summarised in Fig. 4. Current sEV miRNA biomarker information including diagnosis, prognosis, and prediction of chemoresistance in BC is shown in Table 3.

**Table 3:** Small extracellular vesicle miRNA biomarkers in breast cancer diagnosis, prognosis and chemoresistance

Biomarker	Source	Type of purpose	Expression	Feature	Technology	Reference
miR-122-5p	Plasma	Diagnosis	↑	N/A	RT-qPCR	[206]
Let-7b-5p	Plasma	Diagnosis	↓	N/A	RT-qPCR	[206]
miR-101 & miR-372	Serum	Diagnosis	↑	N/A	RT-qPCR	[207]
miR-188-5p	Serum	Diagnosis	↓	N/A	RT-qPCR	[208]
miR-1246	Plasma	Diagnosis	↑	Combination with miR-21 showing better diagnostic value	NGS-Illumina	[209]
miR-21	Plasma	Diagnosis Prognosis	↑	High expression in later-stage BC	NGS-Illumina	[209–211]
miR-7641	Cells/plasma	Diagnosis	↑	Up-regulated in TNBC Related to proliferation & migration	Microarray/RT-qPCR	[212]
miR-9	Cells	Diagnosis	↑	Up-regulated in TNBC Related to metastasis	RT-qPCR	[213]
miR-155	Cells	Diagnosis Chemoresistance	↑	Up-regulated in TNBC Related to DOX and PTX resistance in BC	RT-qPCR	[213, 214]
miR-105	Cells/Mouse	Diagnosis	↑	Up-regulated in TNBC	Solex deep sequencing	[58]
miR-373	Cells/serum	Diagnosis	↑	Up-regulated in TNBC Related to proliferation & migration	RT-qPCR	[207, 215]
miR-223-3p	Plasma	Diagnosis	↑	Related to invasive BC	RT-qPCR	[216]
miR- 338-3p, miR-340-5p & miR-124-3p	Serum	Prognosis	↑	Related to BC recurrence	miRNA PCR array	[217]
miR-29b-3p, miR-20b-5p, miR-17-5p, miR-130a-3p, miR-18a-5p, miR-195-5p, miR-486-5p & miR-93-5p	Serum	Prognosis	↓	Related to BC recurrence	miRNA PCR array	[217]
miR-16 & miR30b	Plasma	Prognosis	↑	Up-regulated in BC with recurrence	Microarray	[218]
miR-93	Plasma	Prognosis	↑	Up-regulated in DCIS	Microarray	[218]
miR-373 & miR-24–2-5p	Plasma	Prognosis	↑	Negatively correlated with survival	NGS-Illumina	[219]
miR-548b-5p, miR-655-3P & miR-376b-5p	Plasma	Prognosis	↓	Positively correlated with survival	NGS-Illumina	[219]
miR-421, miR-128–1 &miR-128–2	Serum	Prognosis	↑	GI-derived three sEV miRNA signature Related to poor prognosis	NGS-Illumina	[202]
miR-100, miR-222, miR-30a & miR-17	Cells	Chemoresistance	↑	Related to DTX resistance in BC	Microarray	[220]
miR-221/222	Cells	Chemoresistance	↑	Related to tamoxifen resistance in BC	RT-qPCR	[221]
let-7a, let-7b, let-7c, miR-103a, miR-16, miR-23a, miR-23b, miR-27a & miR-30a	Cells	Chemoresistance	↑	Related to DTX resistance in BC	Microarray	[222]
miR-130a, miR-20b, miR-25, miR-425, miR-455-3p, miR-4725-5p, miR-551, miR-92	Cells	Chemoresistance	↓	Related to DTX resistance in BC	Microarray	[222]
miR-9-5p, miR-195-5p & miR-203a-3p	Cells	Chemoresistance	↑	Related to DOX and DTX resistance in BC	NGS-Illumina	[223]
miR-378a-3p, miR-378d	Cells	Chemoresistance	↑	Related to DOX and PTX resistance in BC	RT-qPCR	[224]

*Abbreviations*: *BC* Breast cancer, *DCIS* Ductal carcinoma in situ, *DOX* Doxorubicin, *DTX* Docetaxel, *GI* Genomic instability, *NGS* Next-generation sequencing, *N/A* Not applicable, *PTX* Paclitaxel, *RT-qPCR* Reverse transcriptase-quantitative polymerase chain reaction, *TNBC* Triple- negative breast cancer

### sEV miRNA biomarkers in BC diagnosis

Several studies have evaluated distinctive miRNA profiles in sEVs between BC patients and healthy controls to find potential blood-based biomarkers for diagnosis. For example, up-regulated miR-122-5p and down-regulated Let‐7b‐5p were identified [206] in sEVs from BC plasma. In serum sEVs, miR-101 and miR-372 [207] were elevated and miR-188-5p [208] levels were lower in BC patients relative to healthy controls. Hannafon et al. profiled miRNAs in BC plasma-derived sEVs using NGS and found higher levels of miR-1246 and miR-21 in patients compared to healthy controls, and the combination of those miRNAs were a better indicator for BC diagnosis than individual levels [209]. Abundance of miR-7641 [212], miR-9, miR-155 [213], miR-105 [58], and miR-373 [207] was higher in sEVs from TNBC cells compared to human mammary epithelial cells and non-metastatic BC cells, suggesting that sEV miRNA levels may be tissue-type dependent and can be used to differentiate BC subtypes. Elevated miR-7641 and miR-373 was also found in sEVs from TNBC serum and plasma and linked to the proliferation and migration ability of BC cells [207, 212]. Also, miR-223-3p levels were higher in plasma sEVs of invasive ductal carcinoma BC patients when compared to those from ductal carcinoma in situ (DCIS) patients and healthy control subjects, showing potential diagnostic value for invasive BC [216]. All in all, sEV miRNAs are a promising biomarker source for BC early diagnosis and for distinguishing different stages of BC for personalised treatment options.

### sEV miRNA biomarkers in BC prognosis

Evaluating prognosis using sEV-based liquid biopsy is an important component for optimising treatment choices for BC patients. An sEV miRNA profiling study indicated that miR-338-3p, miR-340-5p, and miR-124-3p were up-regulated, and miR-29b-3p, miR-20b-5p, miR-17-5p, miR-130a-3p, miR-18a-5p, miR-195-5p, miR-486-5p, and miR-93-5p were down-regulated in the serum of BC patients with recurrence compared to patients without recurrence [217]. Ni et al. profiled sEV miRNAs in plasma of DCIS patients and primary BC patients with recurrence compared to healthy women [218]. Levels of miR-16 and miR30b were found to be relatively higher in recurrent patients, and miR-93 in DCIS patients [218], demonstrating the promise of sEV miRNAs to distinguish recurrent BC from the early stages of the disease. In another study, 35 differentially expressed miRNAs in plasma sEVs from early-stage BC patients were profiled [219]. Among these miRNAs, highly abundant miR-375 and miR-24–2-5p were found to be negatively correlated with patient survival, and significantly down-regulated miR-548b-5p, miR-655-3P, and miR-376b-5p were found to be positively correlated with survival. Recently, Bao et al. used a combination of genomic instability (GI) analysis with sEV miRNA profiling to identify novel BC biomarkers. In their study, three higher GI-derived sEV miRNA signatures (miR-421, miR-128–1, and miR-128–2) in the serum of BC patients were found to be associated with poor prognosis [202]. These findings support sEV-derived miRNAs as a useful tool for BC prognosis.

### sEV miRNA biomarkers in predicting BC chemoresistance

Deciphering mechanisms of cancer chemoresistance is a critical step for developing or selecting therapeutic agents that bypass resistance and this can also aid in the identification of biomarkers that can be used as predictors of resistance. Chen et al. showed that miR-100, miR-222, miR-30a, and miR-17 were transferred from BC-resistant to -sensitive cells by sEVs and miR-222 reduced expression of the tumour suppressor gene PTEN in sensitive BC cells [220]. Up-regulation of sEV miR-221/222 was also detected in tamoxifen-resistant BC cells [221]. When miR-211/222 sEV levels were reduced, tamoxifen resistance decreased accordingly, showing the potential of using this miRNA as a predictive tool to monitor the progression of tamoxifen-resistant BC.

In addition to these findings, Chen et al. found that sEVs from doxorubicin (DOX)- and docetaxel (DTX)-resistant BC cells had over 300 differentially expressed miRNAs compared to drug-sensitive BC cells [220]. The most abundant sEV miRNAs in DTX-resistant cells were let-7a, let-7b, let-7c, miR-103a, miR-16, miR-23a, miR-23b, miR-27a, and miR-30a, and the most down-regulated miRNAs were miR-130a, miR-20b, miR-25, miR-425, miR-455-3p, miR-4725-5p, miR-551, and miR-92 [222]. These miRNAs are known to modulate target genes involved in transcriptional regulation, protein phosphorylation, kinase activity, and protein binding. Shen et al. also profiled sEV miRNAs in DOX- and DTX-resistant BC cells and reported up-regulation of miR-9-5p, miR-195-5p, and miR-203a-3p that target the one cut homeobox 2 (ONECUT2) transcription factor [223]. Yang et al. reported that serum sEVs from DOX- and PTX-resistant BC patients had higher levels of miR-378a-3p and miR-378d, and suggested a novel mechanism whereby DOX and PTX chemotherapy activated the EZH2/STAT3 pathway in BC cells and led to higher abundance of these miRNAs in serum sEVs [224]. Both of the sEV miRNAs were delivered to neighbouring BC cells to activate the WNT and NOTCH stemness pathways via suppression of Dickkopf 3 (DKK3) and NUMB expression and could induce chemoresistance. Moreover, relatively higher miR-155 abundance was detected in DOX- and PTX-resistant TNBC cell sEVs and the resistance was found to be mediated by translocation of miR-155 via BC-derived sEVs, suggesting that sEV miRNAs could be used as a biomarker for prediction of DOX and PTX resistance and to help guide therapy [213, 214].

### Limitations of sEV miRNA biomarkers in cancer research

It is difficult to orthogonally validate miRNA profiling using microarray and NGS techniques, possibly due to the reduced selectivity of NGS [225]. In addition, miRNA library composition for NGS is dependent on sample library preparation kits. For example, in different individual libraries, the number of detectable sEV miRNAs was found to vary from 380 to 474 [194]. The different miRNA libraries might cause biased NGS data for certain transcripts, but highly abundant miRNAs are less affected by library preparation [226].

Several studies have shown that the choice of methodology for sEV isolation and RNA extraction can significantly affect the abundance of sEV miRNAs and this is also influenced by sample type, resulting in significant differences in miRNA profiling. For example, Park et al. reported that the number of unique miRNA species from urinary sEVs isolated by UC, qEV, and ExoQuick preparations were different, and ExoQuick showed the lowest number of sEV miRNA species compared to the others [227]. However, in blood samples, Kuhlmann et al. demonstrated that ExoQuick provided optimal miRNA yield and consistent small RNA libraries [228]. On the other hand, Buschmann et al. showed that precipitation and membrane affinity-based sEV isolation methods were equally efficient at sEV miRNA profiling, resulting in high absolute numbers of mapped miRNA reads and increased separation of patients group [226]. When six commercial RNA extraction kits were compared, the Norgen Biotek exosome purification and RNA isolation kit had a > fourfold increase of sEV miRNA species in human urine than the other kits [229]. For miRNA biomarker discovery, selection of a high throughput platform, library preparation method, downstream analysis, and sEV isolation and RNA extraction protocol regarding sample type must all be carefully considered.

miRNAs contain heterogenous ends leading to miRNA isoforms (isomiRs) which can affect the precision and accuracy of miRNA profiling and detection [230]. NGS-based sequencing is the only high throughput platform with the ability to detect individual isomiRs but most computational tools do not include isomiR deconvolution by default, so users must consider this option [231, 232]. Some studies have suggested that isomiRs have a role in sorting miRNAs into sEVs and therefore contain discriminatory information which is useful for disease biomarkers [233–235]. Thus, when searching for novel sEV miRNAs and verifying them as cancer biomarkers or therapeutic targets, isomiRs and their functional association with disease should be included the development pipeline.

## Conclusions and future perspectives

To improve BC diagnosis and prognosis, researchers have increasingly focused on liquid biopsy due to its limited invasiveness and suitability for cancer monitoring. According to the evidence described here, monitoring changes of sEV-derived proteins and miRNAs holds great potential and promise for BC biomarker translation. Based on the advances in technology and amount of research described in this review, there are some critical considerations pertaining to sEV-related BC research. Firstly, a combination of approaches might be necessary to accurately diagnose or predict BC. Several lines of recent research have focused on BC multi-omic biomarker signatures that have higher sensitivity for diagnosis and prognosis than a single-omic biomarker. For example, the combination of serum sEV differentiation antagonising non-protein coding RNA (DANCR) and traditional tumour markers CA 15–3 and CEA showed 91.4% specificity and 90.8% sensitivity in BC diagnostic value with improved accuracy over individual biomarkers [236]. Moreover, the combination of serum sEV miR-1910-3p with CA 15–3 was more sensitive than individual biomarkers with 96% diagnostic sensitivity in BC [237]. For early BC patients, combined expression of urinary sEV miR-21 and matrix metalloproteinase-1 (MMP-1) showed improved sensitivity (95%) and specificity (79%) [160]. Additional studies to evaluate multi-omic biomarkers are required.

Secondly, extracellular vesicle and particles (EVPs) are heterogenous nanovesicles and particles classified into subpopulations according to size. In 2018, it was discovered that a new EVP subpopulation called exomeres (smaller than 35 nm), had distinct N-glycan and proteomic profiling compared to other EV subpopulations, and exomeres proteins were associated with glycolysis, metabolic pathways, coagulation, and hypoxia [238]. In 2021, the smallest EVPs named supermeres was reported, and these supermeres showed different RNA and protein profiles relative to exomeres and sEVs [239]. Several tumour-associated proteins and EV-related RNAs were enriched in supermeres [239]. Thus, proteins, glycans, lipids, and nucleic acids might be selectively encapsulated into different EVP subpopulations and have unique biomechanical characteristics and functions in terms of tumour biology. More work is required to thoroughly characterise each EVP subpopulation and better techniques for EVP isolation need to be developed to obtain high-quality samples in a cost and time efficient manner.

Progress has been made but there is still a long road ahead to bring sEV biomarkers from the bench to the bedside. A lack of standardisation in isolation and characterisation obstructs progress, resulting in lower reproducibility and significant inter-study variation. In addition, a limited number of study cohorts, heterogeneous clinical samples, and the complexity of biofluids are additional obstacles to sEV biomarker utility in the clinical setting. Despite these challenges, sEV proteins and miRNAs are promising candidate biomarkers for BC, and research and development for clinical usage are moving forward.

## Data Availability

Not applicable.
